# Effective Population Size Dynamics and the Demographic Collapse of Bornean Orang-Utans

**DOI:** 10.1371/journal.pone.0049429

**Published:** 2012-11-15

**Authors:** Reeta Sharma, Natasha Arora, Benoit Goossens, Alexander Nater, Nadja Morf, Jordi Salmona, Michael W. Bruford, Carel P. Van Schaik, Michael Krützen, Lounès Chikhi

**Affiliations:** 1 Population and Conservation Genetics, Instituto Gulbenkian de Ciência, Oeiras, Portugal; 2 Anthropological Institute and Museum, University of Zurich, Zurich, Switzerland; 3 Organisms and Environment Division, School of Biosciences, Cardiff University, Cardiff, United Kingdom; 4 Danau Girang Field Centre, c/o Sabah Wildlife Department, Kota Kinabalu, Sabah, Malaysia; 5 CNRS, Laboratoire Evolution and Diversité Biologique, Université Paul Sabatier, Toulouse, France; 6 Université de Toulouse, Toulouse, France; University of California, Berkeley, United States of America

## Abstract

Bornean orang-utans experienced a major demographic decline and local extirpations during the Pleistocene and Holocene due to climate change, the arrival of modern humans, of farmers and recent commercially-driven habitat loss and fragmentation. The recent loss of habitat and its dramatic fragmentation has affected the patterns of genetic variability and differentiation among the remaining populations and increased the extinction risk of the most isolated ones. However, the contribution of recent demographic events to such genetic patterns is still not fully clear. Indeed, it can be difficult to separate the effects of recent anthropogenic fragmentation from the genetic signature of prehistoric demographic events. Here, we investigated the genetic structure and population size dynamics of orang-utans from different sites. Altogether 126 individuals were analyzed and a full-likelihood Bayesian approach was applied. All sites exhibited clear signals of population decline. Population structure is known to generate spurious bottleneck signals and we found that it does indeed contribute to the signals observed. However, population structure alone does not easily explain the observed patterns. The dating of the population decline varied across sites but was always within the 200–2000 years period. This suggests that in some sites at least, orang-utan populations were affected by demographic events that started before the recent anthropogenic effects that occurred in Borneo. These results do not mean that the recent forest exploitation did not leave its genetic mark on orang-utans but suggests that the genetic pool of orang-utans is also impacted by more ancient events. While we cannot identify the main cause for this decline, our results suggests that the decline may be related to the arrival of the first farmers or climatic events, and that more theoretical work is needed to understand how multiple demographic events impact the genome of species and how we can assess their relative contributions.

## Introduction

Genetic data are increasingly used to infer the demographic history of natural populations, such as population size changes, gene flow/connectivity between populations and other processes that influenced species over millennia [1], [2], [3]. How present-day patterns can be used to investigate the relative effects of ancient historical processes and recent human-induced changes is at the center of ongoing research [4], [5], [6], [7].

In this context, the last couple of decades have seen an increase in the use of full likelihood-based methods and software to estimate important demographic parameters [2], [3], [8], [9], [10], [11], [12], [13]. Even though these methods provide important insights into the demographic history of the species studied, their power to infer demographic processes in natural populations are still being explored and under much debate, particularly when trying to understand the combined or confounding effect of population size change, structure and fragmentation [14], [15], [16], [17], [18].

Such methods among others have been used to investigate the demographic history of orang-utans, the only Asian non-human great apes. While formerly widespread throughout South and Southeast Asia, orang-utans are currently found as two distinct species surviving in fragmented populations on the islands of Borneo (*P. pygmaeus*) and Sumatra (*P. abelii*) [19]. Reports from early explorers and naturalists in the 19^th^ century suggest that wild orang-utans in these islands were historically encountered at high densities across their distribution range [20].

Four major factors can be invoked to explain the current distribution and population sizes across the whole species ranges. First, climatic changes during the middle to late Pleistocene, which were also accompanied by changes in sea levels, and volcanic eruptions, may have led to major habitat shifts that would have driven orang-utan populations to extinction across vast areas. Contraction within a glacial refugium during the penultimate glaciation was inferred by Arora *et al*., [21] from their phylogenetic reconstruction of Bornean mtDNA haplotypes. This inference was based on the unexpected recent coalescence for all Bornean haplotypes 176 thousand years ago (kya), and the star-shaped tree topology indicative of a recent expansion. A more recent study by Locke *et al*., [22] used genomic data and inferred a decline in Bornean orang-utans after their split from the Bornean-Sumatran ancestral orang-utan population (400 kya). This latter study however did not mention any expansion (as in [21]) or any recent bottleneck (as in [11], see below).

Second, hunting related to the arrival of first hunter-gatherers in Southeast Asia around 40 kya, might have also resulted in severe population size changes. Numerous studies have suggested that orang-utans have been a favorite prey of hunters [23], while their slow development to sexual maturity and long inter-birth interval gives them a very low tolerance to hunting, suggesting that their historical distribution may have been largely determined by past hunting pressures [19].

Third, extensive habitat destruction and hunting of orang-utans following the arrival of early farmers (∼4–5 kya) who may have cleared land for agriculture, could have also affected orang-utan demographic history [24], [25]. We note, however, that there is no direct evidence suggesting that farmers had a strong negative impact on their habitat.

Fourth, habitat loss through recent deforestation for agriculture, logging and fires in the last 150–200 years [20] may have led to dramatic population decline. Rijksen and Meijaard [26] estimated that numbers decreased from 23,000 in 1996 to 15,000 individuals in 1997, a reduction of some 33% in one year due to habitat loss and fires. Currently, most orang-utan populations exist in highly fragmented landscapes [27], [28], and are at high risk of extinction as a result of forest clearance [26], [29], [30], [31], [32].

To examine whether a signature for population decline is found in orang-utan populations and whether this is mainly attributed to ancient or recent events, Goossens *et al*., [11] investigated orang-utan populations from the highly fragmented region of the Lower Kinabatangan (Sabah, northern Borneo). In this region orang-utan populations exist in small forest patches due to recent habitat loss and a consequent concentration of the population in the remaining forests. Using data from 14 microsatellite loci and a series of inferential methods, Goossens *et al*., [11] found a strong signature for a recent collapse in orang-utan populations attributed to anthropogenic factors (deforestation and fragmentation) and not ancient events.

Other studies using different markers, datasets and statistical methodologies, have provided new insights, but these are not always congruent and have as yet not been connected to form a unified framework of orang-utan evolutionary history. The study by Goossens *et al.,* [11] used microsatellite loci and identified a bottleneck signal attributed to recent forest loss. Arora *et al*., [21] used mtDNA and inferred a population contraction followed by expansion throughout the island starting 176 kya. Locke *et al.,* [22] used SNPs and identified a continuous decrease of Bornean orang-utans since their split from Sumatran orang-utans around 400 kya. A potential problem of most testing for population size changes is the failure to account for population structure. Indeed, recent empirical and theoretical studies [14], [16], [33] suggest that population structure can generate spurious signals of population size change, even when populations remained stationary.

**Figure 1:**
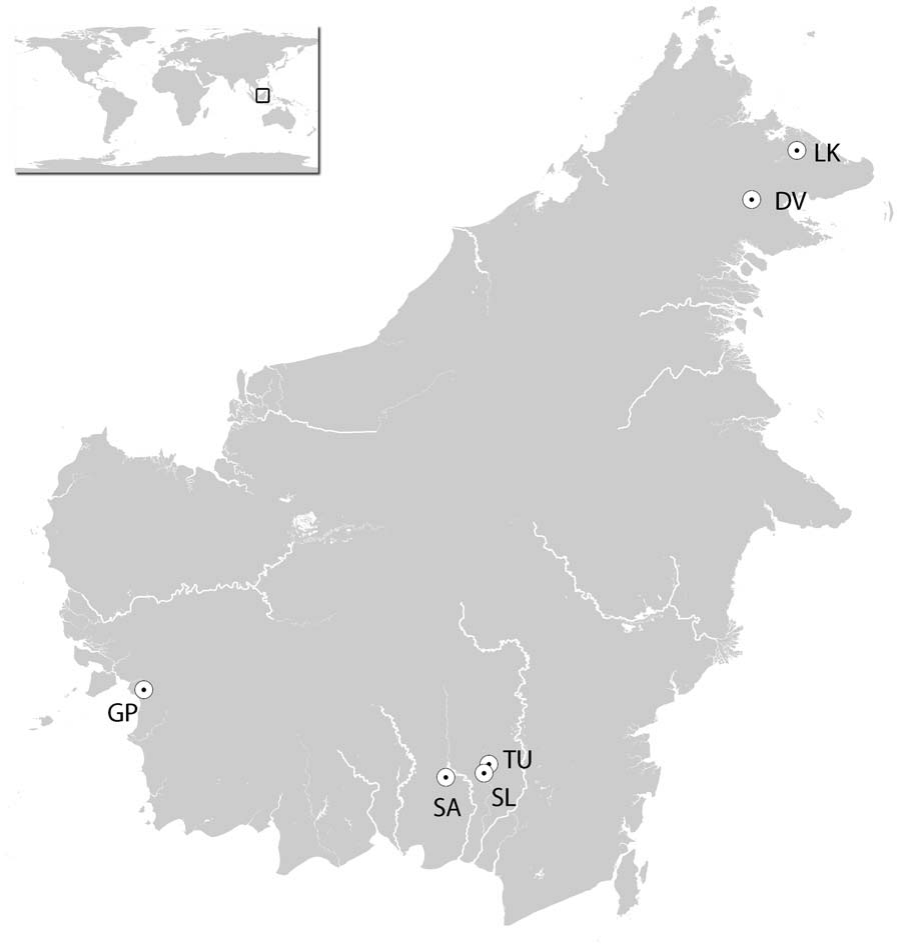
Bornean orang-utan sampling sites. The sample sizes for each site are as follows: DV = Danum Valley (*n* = 20), LK = Lower Kinabatangan (*n* = 26), TU = Tuanan (*n* = 29), SL = Sungai Lading (*n* = 26), SA = Sabangau (*n* = 19), and GP = Gunung Palung (*n* = 6).

In the present study, we investigate the demographic history of Bornean orang-utans, examining population size changes while accounting for population structure. We extend the regional study of Goossens *et al*., [11] by incorporating populations from multiple regions in Borneo (see Figure 1) genotyped by Arora *et al.,* [21]. This allows us to use 126 individuals genotyped at the 12 microsatellite loci used in common in the two previous studies ([21] and [11]). The sampling sites are generally separated by ecological barriers, and present different levels of forest fragmentation. The different demographic histories at each site are expected to have generated different genetic signatures on orang-utan populations. Capitalizing on our extensive data set, we aim to determine whether the signature of recent population decline as found by Goossens *et al.,* [11] is shared by orang-utans across Borneo and is not an artefact of population structure. To do this we use the pooled sample strategy suggested by Chikhi *et al*., [14]. We test whether there potential signal of population size change is ancient or recent, within a framework of four different hypothesis, each corresponding to one of the four major factors that could have led to population decline.

Our results show that: i) all sites exhibit a clear signal for population collapse, ii) population structure alone does not appear to account for the detected signals, and iii) the timing of the population collapse is relatively recent and corresponds to the arrival of first farmers, but varies substantially across all sites.

**Figure 2:**
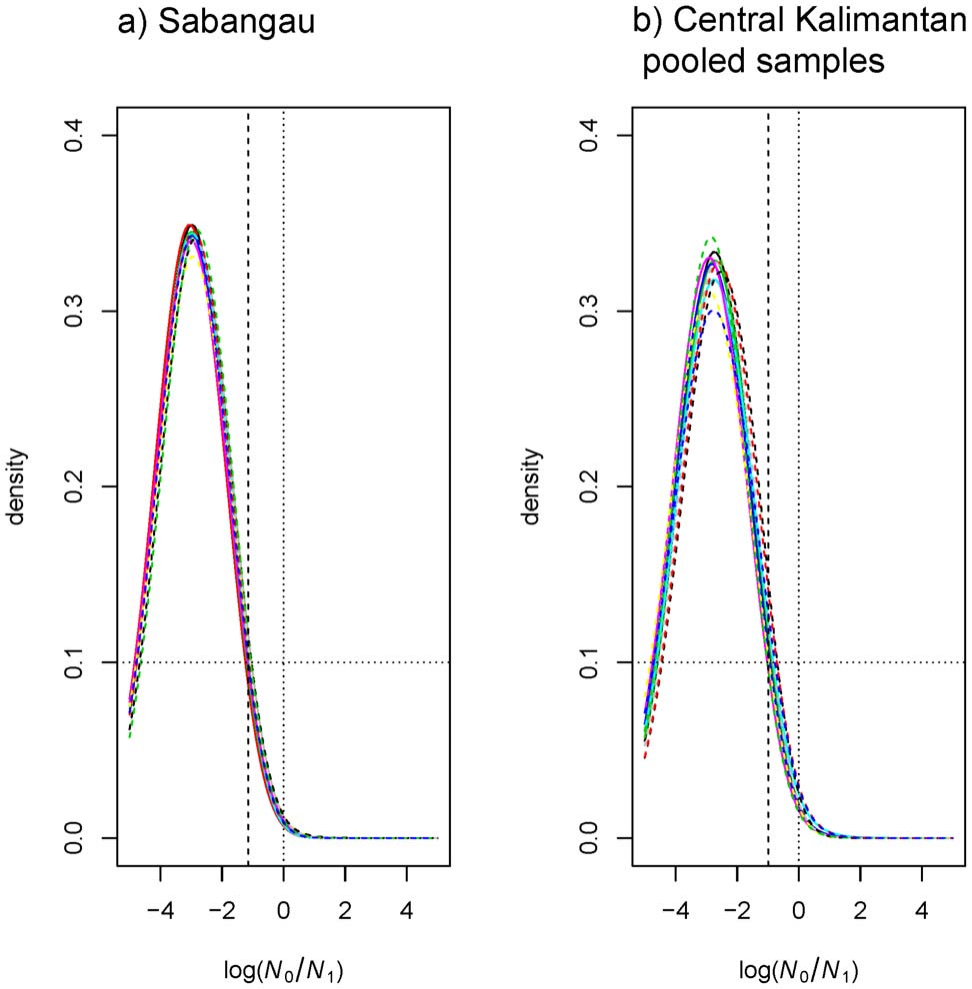
Demographic collapse detected using MSVAR 0.4. Posterior distributions of the effective population size change, log (*N*_0_/*N*_1_). This is shown here for one of the investigated site (Sabangau) in Central Kalimantan and pooled samples analyses from the same region. Note that for illustrative purposes only two plots are shown (see Figure S1 for the other regions). Solid lines (multiple independent runs) correspond to the linear population size change model. Dashed lines (multiple independent runs) correspond to the exponential population size model. Log (*N*_0_/*N*_1_) represents the ratio of present (*N*_0_) to past (*N*_1_) population size. The dotted vertical line corresponds to the absence of population size change, log (*N*_0_/*N*_1_) = 0. The prior distribution is shown for comparison (flat dotted line). The vertical dashed line corresponds to the 95% quantile of the posterior distribution.

**Figure 3:**
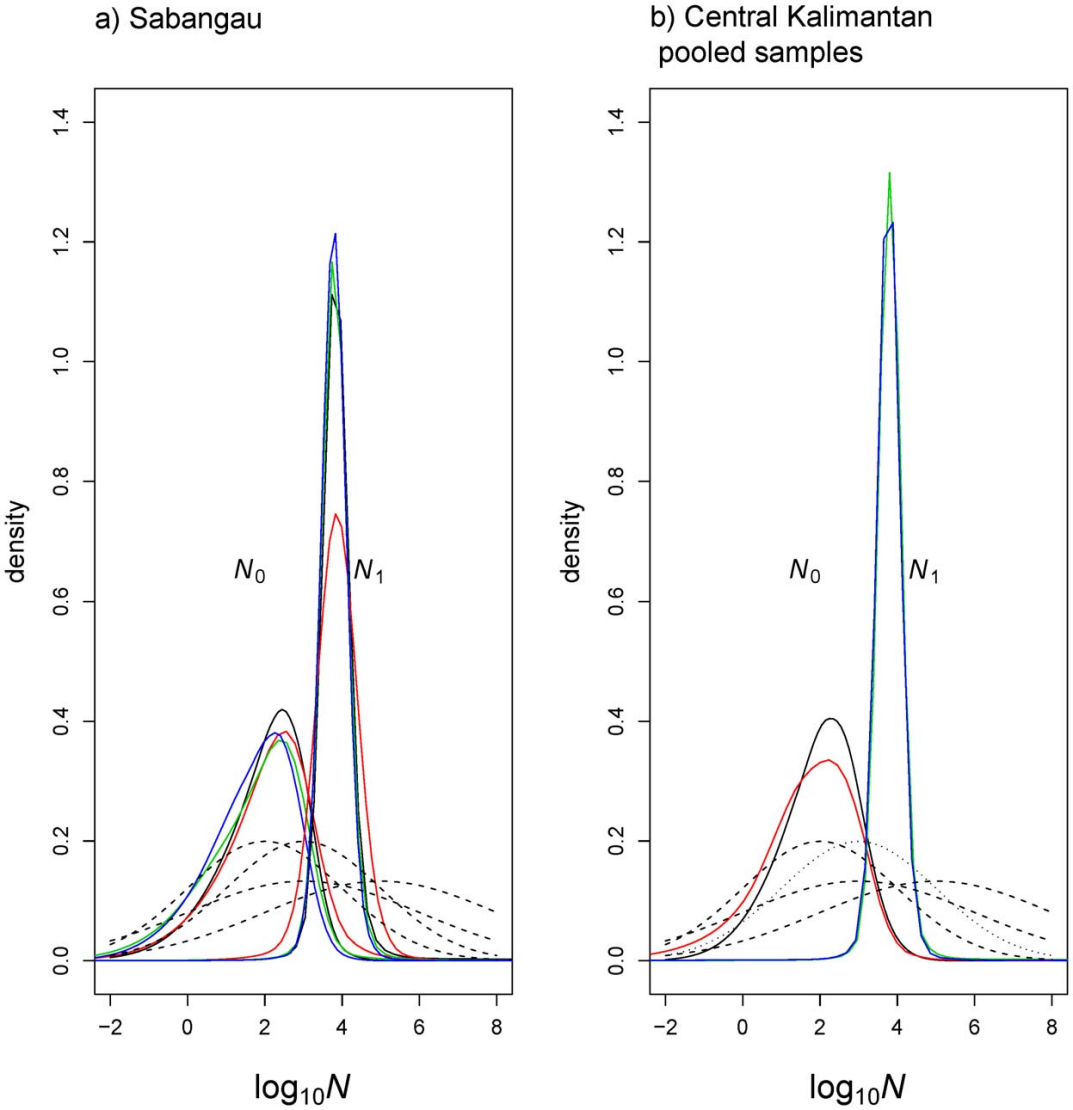
Posterior distributions for the past (*N*_1_) and present (*N*_0_) effective population sizes using MSVAR 1.3. This is shown here for one of the investigated site (Sabangau) in Central Kalimantan and pooled samples analyses from the same region represented on a log_10_ scale. The solid lines correspond to the posterior distribution obtained by independent MCMC runs. Dashed lines correspond to the different priors used for *N*_0_ and *N*_1._

## Materials and Methods

### Study Sites

In this study, we performed a demographic analysis of several populations across the range of the Bornean orang-utans. We capitalized on autosomal microsatellite marker data from 126 individuals genotyped by Goossens *et al*., [31] and Arora *et al*., [21]. As each of these studies amplified a different number of microsatellite markers, we used the twelve markers typed in both of these studies to enable comparison of results. In total, data was available for six study sites, as illustrated in Figure 1: Gunung Palung (hereafter GP, *n* = 6), Sabangau (SA, *n* = 19), Sungai Lading (SL, *n* = 26), Tuanan (TU, *n* = 29), Danum Valley (DV, *n* = 20) and Lower Kinabatangan Lot2 (LK Lot2, *n* = 26) as standardized by Arora *et al*., [21].

### Genetic Analyses

#### Genetic diversity and differentiation

Genetic diversity was measured as the mean number of alleles across loci (MNA), observed heterozygosity (*H*_o_), and unbiased expected heterozygosity (*H*_e_) estimated according to Nei [34]. Departures from Hardy-Weinberg were assessed comparing the observed *F*_IS_ [35] with its distribution after 10,000 permutations of alleles among individuals. The genetic differentiation was measured with pairwise *F*_ST_ [35]. Significant deviations from the null hypothesis of no differentiation were assessed with 10,000 permutations of individuals among populations. These analyses were performed using the Genetix 4.05 software [36].

### Population Size Changes

#### Sampling schemes

We used two different sampling schemes to investigate changes in effective size of Bornean populations: i) examining each individual site, and ii) examining pooled sample sets.

Most population genetic inferences are based on the assumption that samples obtained from one location can be approximated by a Wright-Fisher model. One underlying assumption is that substructure and immigration from other populations can be ignored. Recent simulation work [14], [15] building on earlier theoretical work by Wakeley [33] drew attention to the fact that population subdivision and/or limited gene flow between differentiated sites can falsely generate a signature of population collapse through a strong effect on the genealogy of samples obtained from one location.

Until now very few studies have tried to evaluate the confounding effect of population structure on inferences of population size changes (but see [1] and [7]). One way of assessing the effect of population structure was suggested by Chikhi *et al*., [14] (following the theoretical results of Wakeley [33]) and consists in taking samples from several demes or populations, as this will provide information on the demography of the metapopulation. If a bottleneck is still detected when samples come from several demes, then there is strong indication that the whole metapopulation was subject to a population size change, perhaps through habitat contraction or fragmentation.

To evaluate whether population structure is partly responsible for bottleneck signals detected in the Lower Kinabatangan and Central Kalimantan regions, we also performed analyses by randomly pooling an arbitrary number of genotypes from: (i) the populations in Central Kalimantan region (SA, SL and TU, *n* = 27), and (ii) in the Lower Kinabatangan region including the ten forest fragments (Lot 1 to Lot 10, *n* = 38), respectively. The genotyped data for the Lower Kinabatangan region (Lot 1 to Lot 10) were obtained from Goossens *et al*., [31].

#### The beaumont, and the storz and beaumont methods

Our analyses of population size changes were carried out using two different but complementary approaches as mentioned in Goossens *et al*., [11]: the Beaumont, and the Storz and Beaumont methods. Both methods detect, quantify and date changes in effective population size. These are full-likelihood Bayesian methods that utilize the information from the full allelic distribution in a coalescent-based framework. The methods use the full allelic distribution taking into account the relative size of microsatellite alleles. They are thus expected to be more efficient at detecting population size changes than methods based on summary statistics [37].

The Beaumont [8] method as implemented in MSVAR 0.4 assumes that a stable population of size *N*_1_ started to decrease (or increase) *t*_a_ generations ago to the current population size, *N*_0_. The change in population size is assumed to be either linear or exponential, and mutations are assumed to occur under a SMM, with rate *θ* = 2*N*_0_µ, where µ is the locus mutation rate. Using a Bayesian coalescent-based MCMC approach, the method estimates the posterior probability distributions of; (1) the magnitude of population size change, *r* = *N*_0_/*N*_1_, (2) the time since the population started changing size scaled by *N*_0_, *t*_f_ = *t*_a_/*N*_0_, and (3) the scaled mutation rate *θ* = 2*N*_0_µ. For each sampled population, the analyses were performed both under the linear and exponential models using different parameter configurations, starting values and random seeds. In this study, wide uniform prior distributions were chosen (between −5 and 5 on a log_10_ scale) for log(*r*), log (*θ*), and log (*t*_f_). Positive log(*r*) values, corresponding to a population expansion, were set as the MCMC starting point. Although MSVAR 0.4 allows for quantification of a population increase or decrease, *N*_0_ and *N*_1_ cannot be estimated independently. Similarly, it can only approximate *t*_a_ as a time scaled by *N*_0_, with *N*_0_ remaining unknown. The total number of iterations was always larger than 5×10^9^ with a thinning interval of 5×10^4^. This method was employed first in order to test for a genetic bottleneck under different models of population size changes.

The Storz and Beaumont method [10] implemented in MSVAR 1.3 quantifies the effective population sizes, *N*_0_, *N*_1_ and the time *T* (in generations) since the population size started to change. This method assumes an exponential population size change. Prior distributions for *N*_0_, *N*_1_, *T* and µ were assumed to be lognormal. At least four runs were performed for each sample with a total number of iterations always larger than 4×10^10^ steps. Different sets of priors were used to test their influence on the posteriors, but in most of the runs we set prior means for *N*_0_, *N*_1_, *T* (on a log_10_ scale) with means 4.0, 4.0 and 5.0, respectively; varying the standard deviations between 1 and 5. For *µ* we set a mean of −3.5 with standard deviation of 0.25, so that values for the mutation rate in the region 10^−4^ to 10^−3^ had reasonable support, as widely assumed in demographic analysis (Table S5, [10]).

For both the Beaumont [8] and Storz and Beaumont [10] methods, the first 10% of each independent analysis were discarded to avoid influence in parameter estimation by starting conditions (burn-in period). Convergence of the different chains were visually controlled and tested using Geweke [38] convergence diagnostic. The results of all the four runs were then pooled into one dataset in order to produce larger and more precise samples of the posterior distributions.

#### Recent versus older population size changes

To separate recent from older factors, we further analyzed the posterior distributions obtained for the time *T* since the population change (in generations). In order to express time in years we initially set the generation time of orang-utans to 8 years (y), following Goossens *et al*., [11] and for comparison purposes. However, these authors also used larger values that are in better agreement with field observations throughout different long-term study sites. Several of these studies indicate that 25 years is a more accurate estimate [19], [39], fitting well with a recent estimation of the generation time of the other great apes [40]. Hence, we also performed calculations on that basis to explore the effect of this parameter on dating. This issue is addressed in the discussion section.

Additionally, we assessed the relative probability of recent *versus* ancient events by determining whether the data favored events that were older or more recent than T = 200 years. In practice, the weights of evidence of the hypothesis that time is <200 years vs. >200 years, were assessed using approximate “Bayes factors” (BF), *i.e*. the ratio of the posterior densities of the two alternative hypotheses, over the ratio of the prior densities of the same two alternative hypotheses. BF ≥10 indicates strong support, BF ranging between 3–10 indicates substantial support and BF ≤3 indicates no support [10], [17].

We identified four hypotheses corresponding to the four factors mentioned in the introduction that may have led to a population size decline in Bornean orang-utans. The four corresponding hypotheses are: (i) H1: the decline is recent and attributable to the commercial forest exploitation (FE) as suggested by field studies, occurring in the last 200 years, (ii) H2: the decline started after the arrival of the first farmers (F) but before the very recent forest exploitation, between 5–0.2 kya, (iii) H3: the decline began following the arrival of hunter gatherers (HG) but before the arrival of the first farmers, between 40–5 kya, and (iv) H4: the decline commenced following major climatic changes but before the arrival of HG, between 100–40 kya. The BFs were first computed for each of the four time intervals against all other periods taken together. Since the different hypotheses correspond to time periods of variable sizes, this may bias the results towards one hypothesis over the others. Thus, we also performed a BF analysis by computing BFs for successive 500 y period as in [1] and [7].

## Results

### Genetic Diversity and Differentiation

All microsatellite loci examined were polymorphic with 5 to 13 alleles per locus across all orang-utan populations (Table S1). The mean number of alleles (MNA) per populations ranged between 3.1 in GP (due to low sample size) and 4.8 in TU and in the LK Lot2 population. Average expected heterozygosity (*H*_e_) values across loci were moderately high in all populations and ranged from a minimum of 0.59 in DV to a maximum of 0.69 in LK Lot2, respectively. Average observed heterozygosity (*H_o_*) values ranged between 0.62 in GP to 0.68 in SA. High values of expected heterozygosity were also observed when orang-utan genotypes were pooled, across Borneo (*H*_e_ = 0.73), across Kalimantan (*H*_e_ = 0.70 including Central Kalimantan and GP), and Central Kalimantan (*H*_e_ = 0.69), respectively. Details of genetic diversity estimates for each population are provided in Table S1. A literature review of estimates of genetic diversity in orang-utans from several other locations across Borneo is given in Table S2. After applying Bonferroni correction, none of the populations in our study deviated from Hardy-Weinberg equilibrium. As found by Goossens *et al*., [31] and Arora *et al*., [21], the pairwise *F*_ST_ calculations indicated that all populations were genetically differentiated from each other, with moderate to high *F*_ST_ values ranging between 0.049 and 0.199 (Table S3).

### Population Size Changes

The full-likelihood Beaumont method [8], which assumes the conservative SMM model, consistently supported a clear population decline in all investigated populations under both models of population size change (*i.e.* linear and exponential). The posterior distributions of the effective population size change for all orang-utan populations are shown in Figure 2 and Figure S1. We examined the ratio, *r* of current (*N*_0_) to ancestral (*N*_1_) effective population sizes, where *r = N*_0_/*N*_1._ Across all runs, the median log_10_(*r*), was between −3.32 and −2.28 in all orang-utan populations (Table S4). This indicates a decline in the effective size of all populations of about two to three orders of magnitude and no support for stability (log_10_(*r*) = 0) or expansion (log_10_(*r*)>0). The weight of evidence of the hypothesis for a population decline (Hyp1) vs. a population increase (Hyp2) was assessed using approximate ‘‘Bayes factors’’ (BF), i.e. the ratio of the posterior densities of the two alternative hypotheses, over the ratio of the prior densities of the same two alternative hypotheses. BF calculated for the population decrease hypothesis showed values between 2.7 (TU) and 6.2 (DV) under the exponential model and 2.9 (TU) and 4.9 (DV) under the linear model. Given that BF ranging between 3 and 10 provide substantial support for a hypothesis, our results are strongly indicative of a population decrease under both exponential and linear models, although we also found some sensitivity to model assumptions (Table S4).

To test whether population decline is not an artifact of population structure, we re-analyzed the data by pooling individuals from differentiated sites within regions (cf. [1], [7]). In Borneo, the regions of Central Kalimantan and Lower Kinabatangan each comprise populations that showed moderate to large pairwise *F*_ST_ values. These values indicate variable levels of genetic differentiation, as expected from locations separated by varying geographic distances in a species with patterns of isolation by distance [21], [31] (see Table S3). The posterior distributions in pooled sample analyses were shifted toward the right (i.e. towards less intense bottlenecks), as expected, but still indicated a population decline, although of reduced magnitude. The ratio, log_10_(*r*) ranged between −2.73 to −2.64 (under linear and exponential models of population size change) for the pooled Central Kalimantan samples (Figure 2b). For the Lower Kinabatangan pooled samples, the observed ratio, log_10_(*r*) was between −2.53 to −2.56 (Figure S1e).

Overall, the generally negative values in the individual populations as well as the pooled sample datasets do not support a population increase or a stable population size. Hence, it does appear that population structure alone may not fully explain the bottleneck signal detected in these two different regions in Borneo [14].

The hierarchical Bayesian approach implemented in MSVAR 1.3 [10] showed very limited overlap in the posterior distributions of *N*_0_ and *N*_1_ in all sites, confirming the population decline (Figure 3 and Figure S2). All posterior distributions (solid lines) were similar to each other but very different from the broad prior distributions (dashed lines). This difference (between wide priors and tight posteriors) further supports the inference of population collapse. The BFs calculated for the population decrease hypothesis indicated a change in population size consistent with a substantial support (BF ≥3) for all populations. The posterior distributions of log (*N*_0_) point to small current effective population sizes of orang-utans, with most values for all populations concentrated between approximately 1.35 and 3.15 on a log scale (50% highest posterior density - HPD), corresponding to 22 and 1431 (lowest in the LK Lot2 and highest in SA), respectively. The median values of the posterior distributions of *N*_0_ on a natural scale were approximately 60 to 200. These values are in sharp contrast with log (*N*_1_)_,_ the ancestral population size, where most HPD values for all populations ranged between 3.5 and 4.12 corresponding to 3,100 and 13,335 individuals, respectively. The natural scale median values were between 5,877 and 7,213 individuals (Table S6). For the pooled samples of Central Kalimantan and the Lower Kinabatangan, the natural scale median *N*_0_ values ranged between 400 and 2,984 and for *N*_1_ between 8,082 and 8,601 individuals, respectively.

### Recent Versus Older Population Size Changes

**Figure 4.Time:**
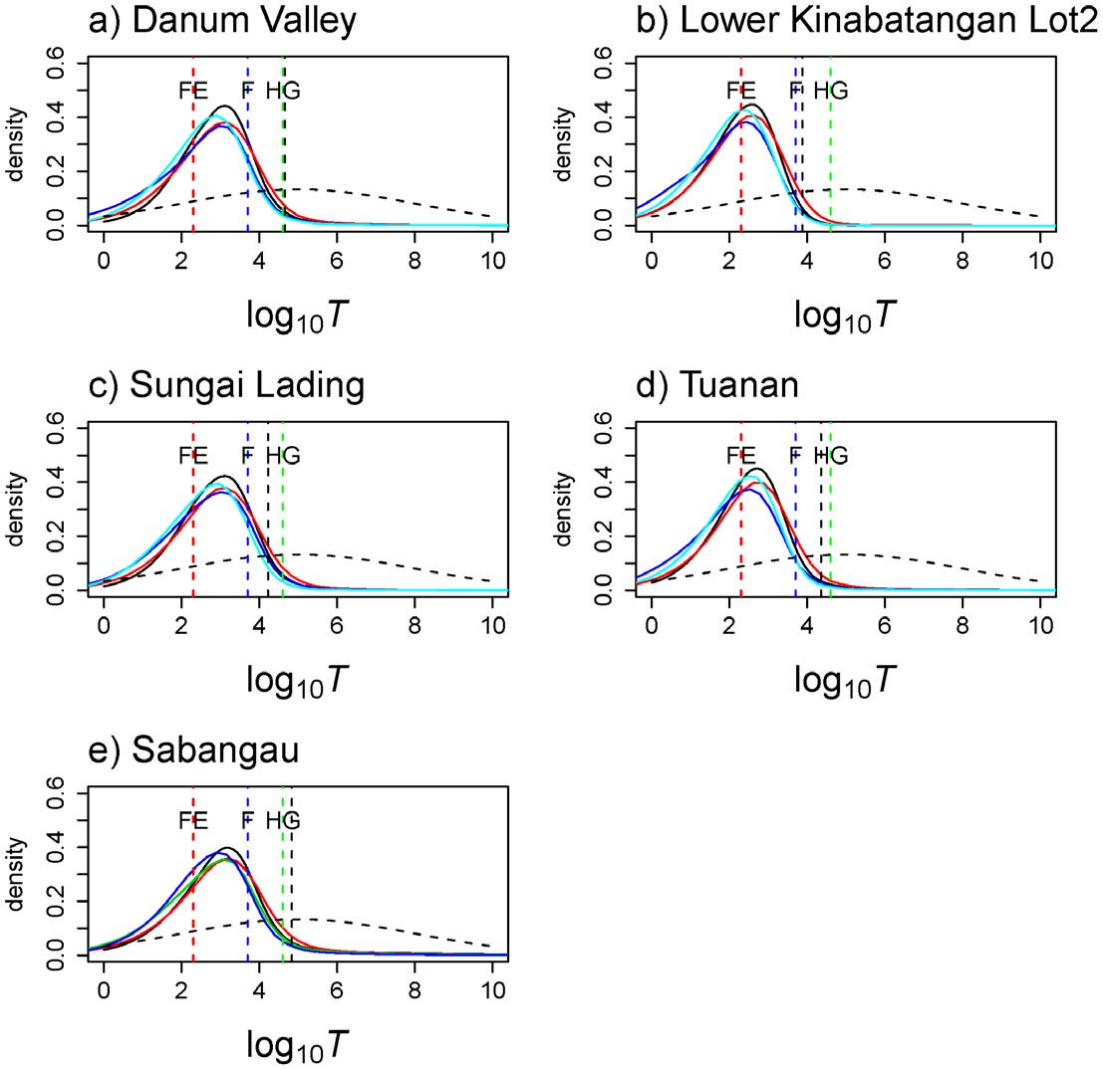
since the population collapse using MSVAR 1.3. The posterior distribution for the time since orang-utan populations (all) collapse started is represented on a logarithmic scale. The different coloured vertical dashed line corresponds to: forest exploitation (FE in red), arrival of farmers (F in blue), and arrival of hunter gatherers (HG in green). The most extreme 95% quantile of the posterior distribution is shown as a black vertical dashed line. The prior is shown as dashed line, its median being 100,000 y ago.

**Figure 5:**
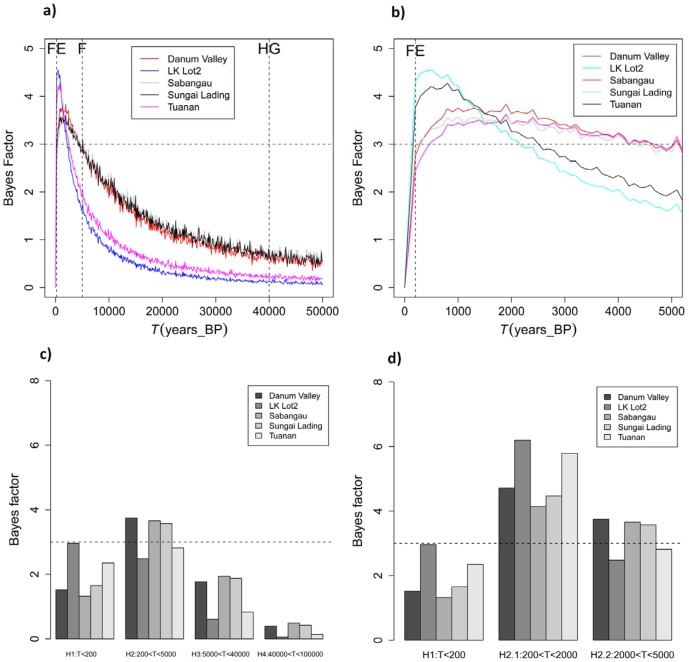
Test of the most likely period associated with orang-utan population decline. Natural logarithms of the Bayes Factors (BF) have been computed to determine during which of the important phases the populations started to decrease. a) The BF values of parameter *T* (time since the beginning of the population collapse) for all orang-utan populations were computed for each 500 year time steps and are plotted for last 50,000 years. The BF value of 3 is plotted as horizontal dash line. The vertical dash lines corresponds to the dates of forest exploitation (FE) ∼ 0.2 kya, arrival of first farmers (F) ∼ 5 kya, and arrival of hunter gatherers (HG) ∼ 40 kya. Note that LK Lot2 is Lower Kinabatangan Lot2 population. b) As 5a), and plotted for the last 5 kya. c) H1 to H4 represents four different hypotheses tested, each corresponding to one of the four factors and time periods that may have led to orang-utan population collapse (discussed in the text). Phase H4 is most ancient and corresponds to time period between 100–40 kya (before the arrival of hunter gatherers (HG) and the time when major climatic and vegetational changes associated with the Pleistocene glaciations occurred). Phase H3 corresponds to the time period after the arrival of HG. Phase H2 corresponds to the time period after the arrival of first farmers (F) and before recent forest exploitation (FE) occurred. Phase H1 is most recent and corresponds to time period of the last 200 years when major forest exploitation occurred in Borneo. Thus, the BF represents the weighted evidence in favor of each phase against the cumulative evidence of all other phases taken together. The dashed horizontal line corresponds to a BF of 3, suggesting that phase H2 has more support than H1, H3, and H4 (see results). d) H2 phase (5,000 years from present) has been divided into two periods: H2.1 and H2.2 which corresponds to the time period just before the recent forest exploitation and after the arrival of the first farmers. The dashed horizontal line corresponds to a BF of 3 and suggesting that phase H2.1 has more support than H1 and H2.2.

The posterior distribution for *T,* the time when the population started to decrease, exhibited median values that were older than the recent forest exploitation (FE) in Borneo, with medians ranging from 358–1,499 years before present (generation time set at 8 years) and 1,188–4,685 (generation time set to 25 years) among all populations, see Table S6, Figure 4 and Figure S3). Also, the pattern of population decline contrasted across sites (see Figure 5a and 5b). While our results indicate a more recent decrease (within last 500 years) in the LK Lot2 (BF = 6.19) and Tuanan (BF = 5.78), the decline appears to be older (>500 years) at the other sites i.e., Danum Valley, Sungai Lading and Sabangau (with BF = 4.70, 4.13, and 4.47, respectively). The estimated 50% HPD limits were broad, ranging between 144 and 9,175 years. The tests of the four hypotheses using the refined BF analysis of *T* posteriors favored the second scenario (H2) (H2, BF>5.1–6.3) over the other three scenarios (0<BF<2.3). Our results suggest that the decline we detected at each site was probably linked to the arrival of the first farmers on Borneo (F). We found little evidence for association of the detected signals with the recent forest exploitation (FE), prehistoric hunting by first hunter-gatherers (HG), or past climatic changes [41], [42] (Figure 5c and 5d). More precisely, the highest BF corresponds to a time period ranging from 0.2 and 2 kya for all populations.

## Discussion

### Genetic Diversity and Differentiation

Across Borneo, we found high levels of genetic diversity (*H*_e_ = 0.73) which suggests that orang-utan populations have been large in the recent past. Such high *H*_e_ values are, as expected, in agreement with a previous study by Goossens *et al*., [31], who analyzed several populations along the Kinabatangan floodplain using 14 microsatellites (including the 12 used here) and found average *H*_e_ ranging between 0.66–0.75. Moreover, we also found significant levels of genetic differentiation, with *F*_ST_ values as high as 0.19 across Borneo. This result is in agreement with limited gene flow resulting from sex-biased dispersal and geographical barriers to dispersal i.e. rivers and mountain chains [21], [31], [43]. Altogether, the levels of genetic diversity and differentiation suggest that populations of orang-utans were probably connected in the recent past, and that recent deforestation has not completely erased patterns of gene flow among populations over millennia.

### Population Size Changes

We found that each of the orang-utan populations assessed exhibited a signal for population decline. Importantly, when we analysed the data using the pooled sampling strategy we found that structure did not fully explain the bottleneck signal. This suggests that orang-utan populations have indeed been subjected to a population contraction in the recent past.

Interestingly, our results also show regional variation in the intensity and timing of the bottlenecks experienced by the populations. For instance, the posterior distribution of *N*_0_ and *N*_1_ showed a 35 to 46-fold decline in Sabangau, Danum Valley and Sungai Lading (using the medians), whereas higher values were observed (between 71 and 111-fold declines) in Tuanan and the Lower Kinabatangan Lot2. Similarly, we obtained a much more recent dating for Lower Kinabatangan Lot2 and Tuanan compared to the other locations (Figure 5a and 5b). Such variation is nonetheless expected as each site has probably experienced a different demographic history.

Despite the inter-site variation, it is striking that for all populations the data point towards a contraction that took place in the last 2,000 years and that is difficult to explain by population structure alone. This raises the question of what may have generated this signal in the genetic data. We first review the historical factors that potentially affected orang-utan populations and then discuss several issues related to population genetic inference.

### On the Potential Causes of Population Size Changes

Our results provide strong support for orang-utan populations decline following the arrival of first farmers. Nonetheless, we cannot exclude the possibility that other major historical events or phenomena might have impacted orang-utan populations. First, climate change in the late Pleistocene, including the last glacial maximum (∼21–18 kya), might also have adversely affected orang-utan populations. Several independent lines of evidence indicate that a series of climatic changes profoundly influenced the geography and vegetation in many parts of Sundaland, leading to shifts in the extension and distribution of different habitat types [44], [45], [46], [47], [48]. These biogeographical changes are thought to have had a profound impact on the structure and geographic distribution of the Sunda Shelf primate fauna, including orang-utans [44], [49].

Second, prehistoric hunting of orang-utans by hunter-gatherer societies most likely started in the late Pleistocene (∼40 kya) [41], [50], [51]. The evidence comes from the recovery of orang-utan remains (comprising more than 30% of non-human primate specimens) from Niah Cave, an archaeological site in Sarawak, and several authors have suggested that the hunting pressure might have been high, causing the local extinction of orang-utans in the wide dry forest areas of Borneo [19], [26]. However, controversy exists regarding the impact of hunting which is difficult to evaluate. For instance, Harrison [41] argued that hunting may have had little impact on Eurasian mega fauna as humans and wildlife coexisted for hundreds of years in both Eurasia and Africa. It is however clear that orang-utans have disappeared from mainland Asia and from most of the islands in Southeast Asia, with the exception of Borneo (the third largest island in the world) and Sumatra, where they survive in remote areas.

Third, recent deforestation has most certainly affected orang-utans, though we may not be able to detect the impact in levels of genetic diversity or differentiation, or date such recent factors.

Indeed, it is also possible that all factors combined have shaped the history of present-day populations. That is, even though we identify a relatively recent collapse within the last <2000 years), it is still possible that this signature of a population decline is a result of more than one contraction occurring at different time periods. The mixed effect of several events on the genetic signatures found in populations has been described before. For instance, in their study of African elephants, Okello *et* *al*., [52] found evidence for the impact not only of heavy poaching in the 1970s but also for events from the mid-Holocene (about 4000 years ago), although the timing of population decline was dated to ∼ 2500 years before present. The authors concluded that when there was a strong effect of both climatic fluctuations and poaching upon the population, the estimated time of population decline identified with MSVAR would not coincide with any of the documented events, but rather at some time between them. In the case of Bornean orang-utans, we may be in a similar situation, where different sites have been impacted in different ways by recent deforestation and farming, as well as ancient hunting and climatic changes.

### Modelling Assumptions and Population Genetic Inference

In the methods used here the dating obtained depends on two main factors. The first is the generation time used. The second is the mutation model including the mutation rate priors used.

Goossens *et al.,* [11] used a generation time of 8 y, which is the time of first reproduction for female orang-utans in the Kinabatangan floodplain. However, the authors also used double and triple values to examine the robustness of their inferences, and found no major difference in the main conclusions. Here we used both the values of 8 y (to compare with Goossens *et al*., [11] study) as well as 25 y [39]. Using either of these two values yields a time of population collapse (details given in Table S6) between the arrival of first farmers and recent deforestation.

It is however important to note that the issue regarding the mutation model and mutation rate typically plagues the dating using population genetic data (this is less of an issue for phylogenetic studies). In the case of orang-utans, this includes the dating obtained by Locke *et al.,* [22] using genomic data. Locke *et al*., [22] found a very recent split between Bornean and Sumatran orang-utans (400 kya) whereas Arora *et al.,* [21] found extremely deep mtDNA split, on the order of 2.3 to 5 million years. Given that mtDNA has a smaller effective size than nuclear markers, the discrepancy between these two studies may be partly due to how mutation rates are estimated for the different markers among other factors that still need to be clarified.

Another issue regarding the posteriors obtained here is that they are still rather wide. More markers would provide more precise estimates on the dating. For instance, Goossens *et al.,* [11] study used 14 loci to analyze one of the populations investigated here (LK Lot2). They obtained a more recent and precise dating of the population decline. However, when we re-analyzed the same population using 12 loci we get wider posteriors for the time estimates. This tends to favor older events.

Also, most of the inferential methods, including the Beaumont [8] method have been developed to account for only a single demographic event, i.e. a single bottleneck or an expansion [3], [10], [17]. It is not clear how parameter estimates are affected when populations have undergone more than one major demographic event, as expected for orang-utan populations from other studies [21], [53].

In addition, there are other complicating factors that may need to be considered. For instance, it was recently found that the social structure of orang-utans in Tuanan consists of many related females and immigrant males [54]. At this stage it is difficult to say how the use of samples from related individuals and inferences of population size change may interact. Furthermore, genetic data from a more diverse array of co-distributed megafauna from similar regions in Southeast Asia should provide a comparative framework and depict a clearer picture of orang-utan population dynamics, to better explain their response to climate change and human activities,.

### Conclusion

Using a large dataset of orang-utans from several independent Bornean populations, we have shown that Bornean orang-utans underwent at least one population collapse, and that this is not due to confounding effect of population structure. Furthermore, we also provide support for a link to the arrival of farming. Nonetheless, we cannot exclude the possibility that other events have generated this genetic signature. Indeed, it is probably that a combination of factors has shaped the signal of population decline observed. To further explore the contribution of the other casual factors and the timing of the consequent demographic events, further efforts are needed to test more complex and realistic demographic models. For more complex models, full likelihood inferential methods may not be the most appropriate and methods based on summary statistics may represent the best option [55]. Using an Approximate Bayesian Computation (ABC) approach, genetic data sets may be simulated for a specified set of scenarios of climatic and/or human-induced events [56]. This approach should allow us to test whether a complex model can explain the past demography of orang-utans better than a simple one as applied in all the studies done so far [11], [21], [22]. The new findings could be used to complement the general view of the demographic history of orang-utans and underscore the need to expand the conservation measures suggested earlier for orang-utan populations in the Lower Kinabatangan region (such as protection of habitat units, elimination of hunting etc.) to the whole species [32].

## Supporting Information

Figure S1 **Demographic collapse detected using MSVAR 0.4.** Posterior distributions of the effective population size change, log (*N*_0_/*N*_1_) for all other orang-utan populations analysed (separate and for pooled samples). Solid lines (multiple independent runs) correspond to the linear population size change model. Dashed lines (multiple independent runs) correspond to the exponential population size model. Log (*N*_0_/*N*_1_) represents the ratio of present (*N*_0_) to past (*N*_1_) population size. The vertical dotted line corresponds to absence of population size change, log (*N*_0_/*N*_1_) = 0. The prior distribution is shown for comparison (flat dotted line). The vertical dashed line corresponds to the 95% quantile of the posterior distribution.
(TIFF)

Figure S2 **Posterior distributions for the past (** ***N*** **_1_) and present (** ***N*** **_0_) effective population sizes using MSVAR 1.3.** This is shown here for all other orang-utan populations represented on a log_10_ scale. The solid lines correspond to the posterior distributions obtained by multiple independent runs. Dashed lines correspond to the different priors used for *N*_0_ and *N*_1._ Note that LK Lot2 is Lower Kinabatangan Lot2 population.
(TIFF)

Figure S3 **Time since the population collapse using MSVAR 1.3.** Posterior distributions for the time since orang-utan populations collapse in years (*T*) represented in log_10_ scale for the pooled samples using 8y generation time. The solid lines correspond to the posterior distributions obtained by multiple independent runs. The different coloured vertical dashed line corresponds to, forest exploitation, FE (in red), arrival of farmers, F (in blue), arrival of hunter gatherers, HG (in green), and 95% quantile of the posterior distribution (in black).
(TIFF)

Table S1 **Genetic diversity of orang-utan populations.** Average number of alleles across all populations for each loci (Na), observed (*H*_o_) and expected (*H*_e_) heterozygosity and Mean number of alleles (MNA) for each populations across 12 microsatellite loci.
(XLSX)

Table S2 **A literature review of estimates of genetic diversity in orang-utans from several locations across Borneo.**
(XLSX)

Table S3 **Estimated pairwise** ***F*** **_ST_ values (below diagonal) and their significance (above diagonal).**
(DOCX)

Table S4 **Posterior values of the bottleneck signal obtained with MSVAR 0.4.**
(XLSX)

Table S5 **Starting values for MSVAR 1.3.**
(XLSX)

Table S6 **Posterior values of the bottleneck signal obtained with MSVAR 1.3.**
(XLSX)
