# Supplementary figures and images for: Effective Population Size Dynamics and the Demographic Collapse of Bornean Orang-Utans
Source: PLoS One. 2012 Nov 15;7(11):e49429. doi: 10.1371/journal.pone.0049429 (PMC3499548; doi:10.1371/journal.pone.0049429)

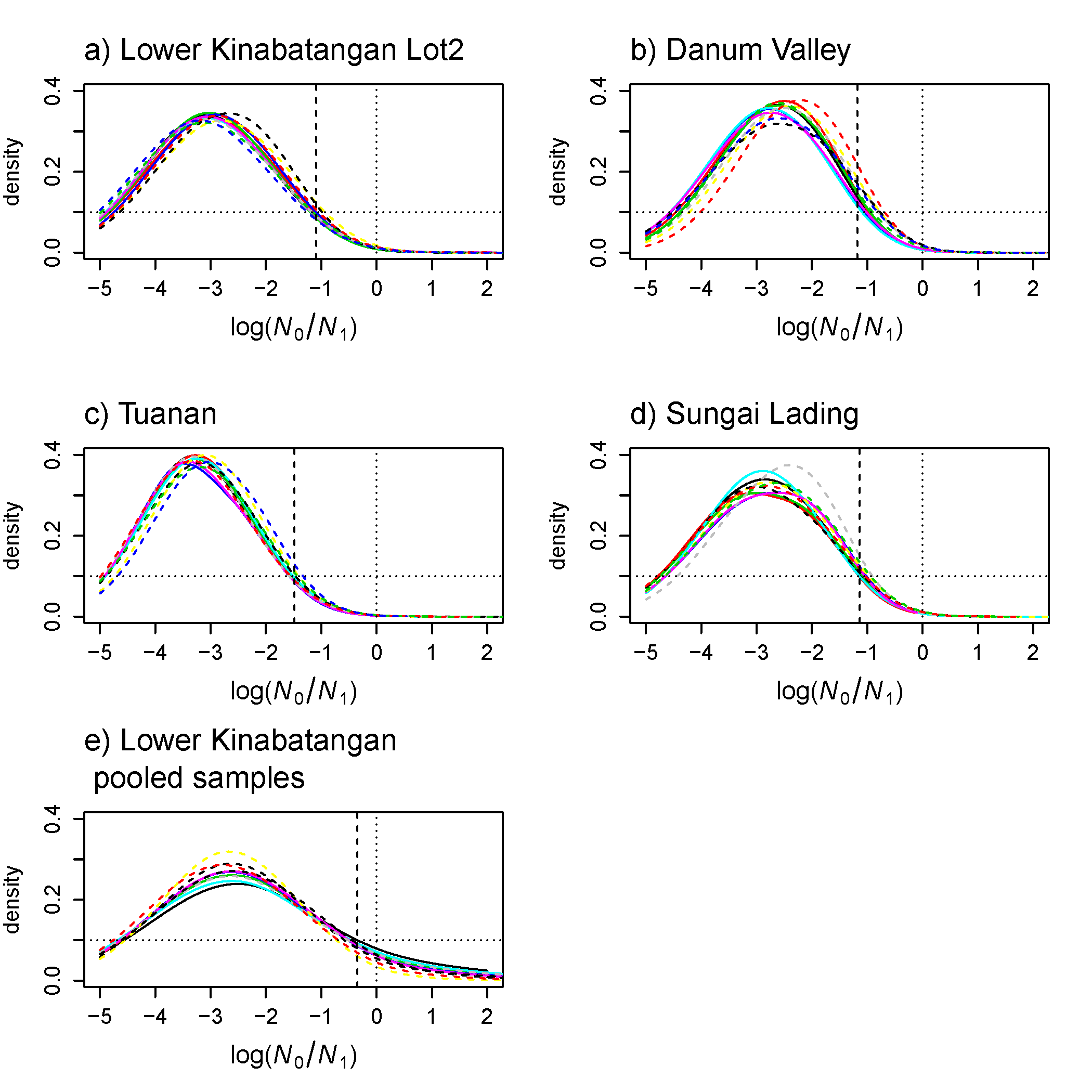

Supplement: Figure S1 — Demographic collapse detected using MSVAR 0.4. Posterior distributions of the effective population size change, log (N 0/N 1) for all other orang-utan populations analysed (separate and for pooled samples). Solid lines (multiple independent runs) correspond to the linear population size change model. Dashed lines (multiple independent runs) correspond to the exponential population size model. Log (N 0/N 1) represents the ratio of present (N 0) to past (N 1) population size. The vertical dotted line corresponds to absence of population size change, log (N 0/N 1) = 0. The prior distribution is shown for comparison (flat dotted line). The vertical dashed line corresponds to the 95% quantile of the posterior distribution. (TIFF) [file pone.0049429.s001.tiff]

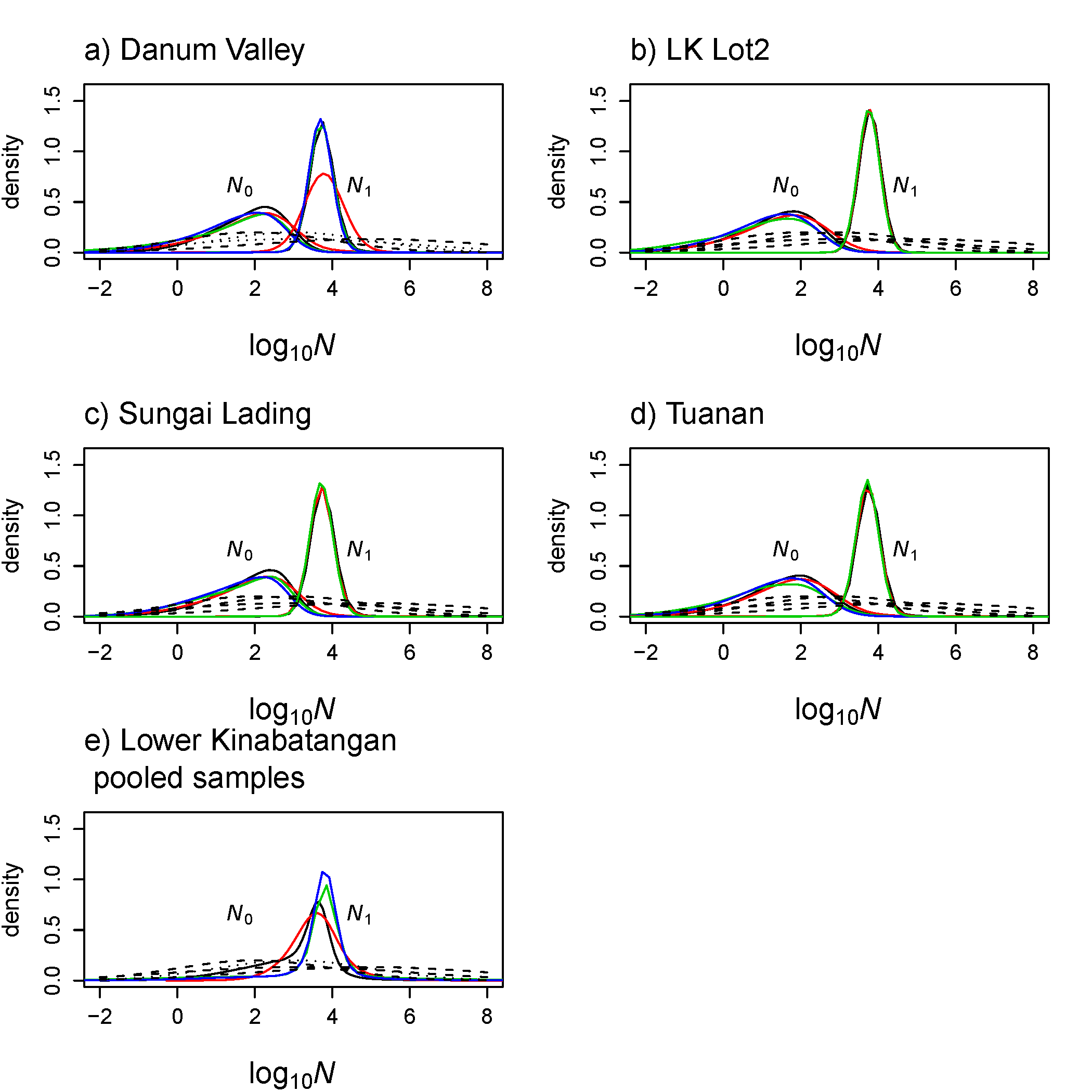

Supplement: Figure S2 — Posterior distributions for the past ( N 1) and present ( N 0) effective population sizes using MSVAR 1.3. This is shown here for all other orang-utan populations represented on a log10 scale. The solid lines correspond to the posterior distributions obtained by multiple independent runs. Dashed lines correspond to the different priors used for N 0 and N 1. Note that LK Lot2 is Lower Kinabatangan Lot2 population. (TIFF) [file pone.0049429.s002.tiff]

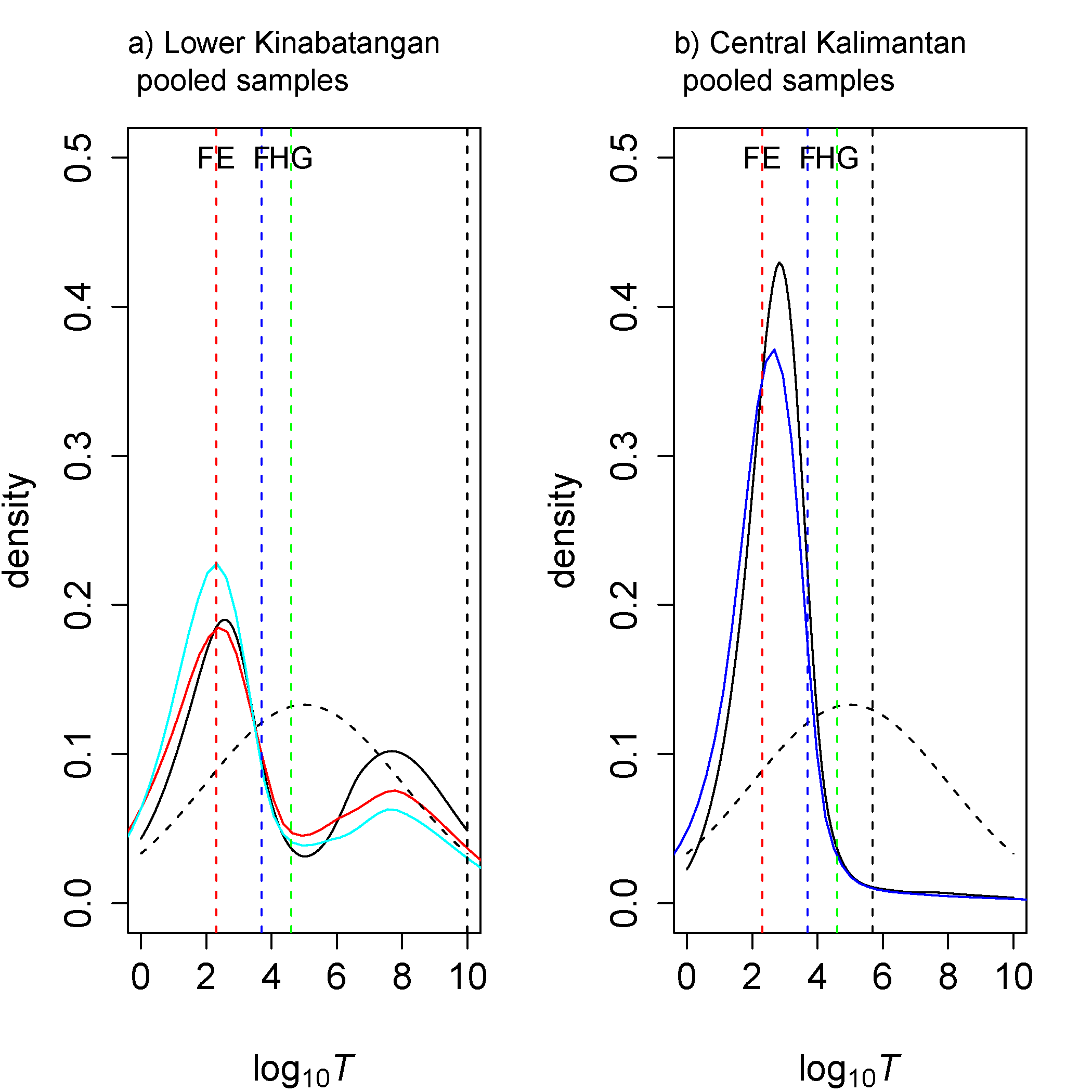

Supplement: Figure S3 — Time since the population collapse using MSVAR 1.3. Posterior distributions for the time since orang-utan populations collapse in years (T) represented in log10 scale for the pooled samples using 8y generation time. The solid lines correspond to the posterior distributions obtained by multiple independent runs. The different coloured vertical dashed line corresponds to, forest exploitation, FE (in red), arrival of farmers, F (in blue), arrival of hunter gatherers, HG (in green), and 95% quantile of the posterior distribution (in black). (TIFF) [file pone.0049429.s003.tiff]
