# Supplementary material for: Effective Population Size Dynamics and the Demographic Collapse of Bornean Orang-Utans
Source: PLoS One. 2012 Nov 15;7(11):e49429. doi: 10.1371/journal.pone.0049429 (PMC3499548; doi:10.1371/journal.pone.0049429)
Supplement: Table S3 — Estimated pairwise F ST values (below diagonal) and their significance (above diagonal). (DOCX) [file pone.0049429.s006.docx]

|  | TU | SL | SA | GP | DV | LK Lot2 |
| --- | --- | --- | --- | --- | --- | --- |
| TU | - | *** | * | *** | *** | *** |
| SL | 0.084 | - | *** | *** | *** | *** |
| SA | 0.049 | 0.110 | - | ** | *** | *** |
| GP | 0.151 | 0.145 | 0.130 | - | * | *** |
| DV | 0.117 | 0.189 | 0.152 | 0.199 | - | *** |
| LK Lot2 | 0.125 | 0.160 | 0.082 | 0.137 | 0.144 | - |

*p<0.05, **p<0.01, ***p<0.001

TU: Tuanan, SL: Sungai Lading, SA: Sabangau, GP: Gunung Palung, DV: Danum Valley, LK Lot2: Lower Kinabatangan Lot2.
